# A Systematic Review of Long-Term Use of Proton Pump Inhibitors (PPIs) in Older Adults on Polypharmacy: Do PPIs Deplete Nutrients?

**DOI:** 10.7759/cureus.90888

**Published:** 2025-08-24

**Authors:** Muhammad Salman Shahid, Nouman Ahmed, Zeeshan Kamal, Laibah Nathaniel, Bhavna Singla, Shivam Singla, Sunita Kumawat, Munaza Batool, Osatohanmwen Ekomwereren, Nabila N Anika, Muhammad Sahil

**Affiliations:** 1 Internal Medicine, King Edward Medical University, Lahore, PAK; 2 Internal Medicine, Mayo Hospital, Lahore, PAK; 3 Internal Medicine, Jinnah Medical College Peshawar, Peshawar, PAK; 4 Internal Medicine, Erie County Medical Center, Buffalo, USA; 5 Internal Medicine, TidalHealth Penninsula Regional, Salisbury, USA; 6 Internal Medicine, Hackensack Meridian Health Ocean University Medical Center, Brick Township, USA; 7 Internal Medicine, Rawalpindi Medical University, Rawalpindi, PAK; 8 Trauma and Orthopaedics, Royal Shrewsbury Hospital, Shrewsbury, GBR; 9 General Surgery, Baylor College of Medicine, Houston, USA; 10 Medicine and Surgery, Holy Family Red Crescent Medical College and Hospital, Dhaka, BGD

**Keywords:** calcium, cognitive decline, iron, magnesium, micronutrient deficiency, nutritional screening, older adults, polypharmacy, proton pump inhibitors, vitamin b12

## Abstract

Proton pump inhibitors (PPIs) are widely prescribed in older adults, often beyond recommended durations, raising concerns about nutrient depletion. This systematic review examined the impact of long-term PPI use (≥6 months) on micronutrient status in older adults receiving polypharmacy. A comprehensive search of PubMed, Scopus, Web of Science, and Cochrane Central Register of Controlled Trials (CENTRAL) identified five eligible studies, including 693 participants. Results showed a 12-18% reduction in serum vitamin B12 over 12 months of PPI use. Calcium and parathyroid hormone levels declined significantly in a 12-month cohort, while bone turnover markers increased despite stable bone mineral density. Findings for magnesium were inconsistent, with results ranging from no change after 12 months to pharmacokinetic alterations without systemic depletion. Overall, the evidence consistently supports an association between prolonged PPI therapy and reductions in vitamin B12 and calcium, with conflicting results for magnesium. These deficiencies may contribute to cognitive decline, bone fragility, and increased fall risk in older adults. Routine nutritional monitoring, targeted supplementation, and deprescribing where appropriate should be considered to mitigate these risks, while further large-scale trials are needed in frail geriatric populations.

## Introduction and background

Proton pump inhibitors (PPIs) are among the most widely prescribed pharmacologic agents worldwide, primarily used for managing acid-related gastrointestinal disorders such as gastroesophageal reflux disease (GERD), peptic ulcer disease, Zollinger-Ellison syndrome, and non-ulcer dyspepsia [1]. Their mechanism of action-irreversible inhibition of the hydrogen-potassium ATPase pump in gastric parietal cells-effectively suppresses gastric acid secretion, providing symptomatic relief and promoting mucosal healing. Due to their efficacy and favorable short-term safety profile, PPIs have become a mainstay in clinical practice, especially in older populations where gastrointestinal disorders are prevalent [2]. However, emerging evidence suggests that chronic use is associated with adverse outcomes, including increased fracture risk, renal disease, and potential cognitive decline, which highlights the importance of balancing their well-established benefits with potential long-term risks [2].

The widespread and often prolonged use of PPIs in older adults has raised growing concerns in recent years. Many patients continue PPI therapy beyond the recommended treatment duration without periodic reassessment of clinical need, a phenomenon known as "inappropriate chronic use" [2]. This is particularly troubling in geriatric populations, where the long-term safety of PPIs becomes more relevant given age-related physiological changes, higher medication burdens, and increased susceptibility to adverse drug events. One of the most pressing concerns involves the potential for micronutrient depletion resulting from sustained gastric acid suppression [3].

Gastric acid plays an essential role in the digestion and absorption of several key micronutrients. By lowering the stomach’s acidity, PPIs may interfere with the ionization, solubilization, and transport mechanisms necessary for optimal uptake of nutrients such as iron, vitamin B12, calcium, magnesium, and folate [4]. Clinical studies have shown that vitamin B12 deficiency occurs in up to 20% of long-term PPI users, hypomagnesemia is well-documented though less frequent, and calcium malabsorption has been linked to higher fracture rates in older adults [4]. The inhibition of intrinsic factor function and gastric acid secretion can reduce vitamin B12 absorption, while calcium and magnesium require acidic conditions for intestinal absorption. Over time, this biochemical disruption may lead to subclinical or overt deficiencies, posing significant clinical implications [4].

Older adults are especially vulnerable to such nutritional disturbances. With advancing age, gastric acid secretion naturally declines, and physiological absorption efficiency decreases. This vulnerability is further magnified by comorbid conditions (e.g., diabetes, chronic kidney disease), reduced dietary intake, and polypharmacy-the concurrent use of multiple medications [5]. Polypharmacy can intensify drug-nutrient interactions, lead to malabsorption syndromes, or potentiate adverse effects from nutrient deficiencies. Furthermore, certain medications, often co-prescribed in this demographic, such as metformin and diuretics, independently impair the absorption of key micronutrients and may have synergistic effects when combined with PPIs [5]. These concerns underscore the need for clinical vigilance, with several guidelines now recommending periodic monitoring of vitamin B12, magnesium, and calcium status in patients on chronic PPI therapy, along with consideration of supplementation or deprescribing strategies when appropriate [5].

Micronutrient deficiencies, especially of vitamin B12, calcium, and magnesium, have been linked to serious geriatric syndromes, including cognitive decline, neuropathy, impaired bone mineralization, increased fracture risk, muscle weakness, and falls [6]. These outcomes not only impact morbidity and mortality but also significantly reduce quality of life and increase healthcare costs in aging populations. Although several observational studies have reported associations between long-term PPI use and nutrient deficiencies, the evidence from prospective trials and randomized controlled studies remains fragmented. Moreover, few syntheses have focused specifically on older adults receiving polypharmacy, a group at particularly high risk. A focused, evidence-based analysis is necessary to clarify the clinical impact of chronic PPI therapy on nutrient absorption and guide informed prescribing, deprescribing, and monitoring practices.

Therefore, the objective of this systematic review is to evaluate evidence from clinical trials and prospective studies regarding the impact of long-term PPI use on serum or plasma micronutrient levels in older adults undergoing polypharmacy. This review aims to identify the most affected nutrients, assess the clinical implications of their deficiencies, and provide actionable insights for optimizing care in this vulnerable population.

## Review

Materials and methods

Study Design and Registration

This systematic review was designed to evaluate the impact of long-term proton pump inhibitor (PPI) use on micronutrient status in older adults receiving polypharmacy. The study was conducted in accordance with the Preferred Reporting Items for Systematic Reviews and Meta-Analyses (PRISMA) 2020 guidelines [7] to ensure methodological rigor and transparency. Although the protocol was not prospectively registered in the International Prospective Register of Systematic Reviews (PROSPERO), all standard steps of systematic review methodology were followed, including structured literature search, duplicate screening, and quality assessment. The decision not to register was primarily due to the limited scope and rapid timeline of the project, which was undertaken as part of a collaborative academic initiative rather than a large-scale funded study. Nonetheless, full methodological transparency has been maintained by adhering strictly to PRISMA guidelines.

Eligibility Criteria and PICO (Population, Intervention, Comparison, Outcome) Framework

The review was guided by a clearly defined PICO framework [8]. The population (P) consisted of adults aged 45 years and above, with the lower cut-off chosen to capture midlife participants who are at risk of polypharmacy, while “older adults” in the context of this review specifically refers to those aged ≥60 years. The intervention (I) was long-term PPI therapy, defined as use for ≥6 months, with or without co-administration of other medications. Studies were included regardless of the specific type of PPI used. The comparison (C) group, when available, included individuals not receiving PPIs or receiving a placebo or other acid-suppressive therapies. The primary outcomes (O) of interest were changes in serum or plasma levels of micronutrients including, but not limited to, vitamin B12, folate, magnesium, calcium, and related biomarkers such as parathyroid hormone (PTH) and bone turnover markers. Both interventional and prospective observational studies were eligible if they provided quantitative assessments of nutrient levels in the context of PPI use. Exclusion criteria included retrospective studies, case reports, reviews, pediatric populations, and studies without direct biochemical assessment of nutrient levels, to ensure focus on high-quality, clinically relevant data.

Eligible studies were limited to those published in English, involving human subjects, and reporting clinical trial or prospective cohort data. Studies were excluded if they were retrospective in nature, did not involve micronutrient assessment, or lacked a clearly defined older adult population. Single-case reports, editorials, reviews, and non-peer-reviewed articles were also excluded.

Literature Search Strategy

A comprehensive literature search was conducted using PubMed, Scopus, Web of Science, and the Cochrane Central Register of Controlled Trials (CENTRAL) to maximize sensitivity and ensure comprehensive coverage of the relevant literature. The search terms included combinations of keywords and Medical Subject Headings (MeSH) such as “proton pump inhibitors”, “PPIs”, “micronutrient deficiency”, “vitamin B12”, “iron”, “magnesium”, “calcium”, “nutritional status”, and “older adults”. Boolean operators were used to refine the results, and filters were applied to include clinical trials and prospective studies published within the last 10 years. Reference lists of included articles were manually screened to identify any additional eligible studies not captured in the electronic search.

Study Selection and Data Extraction

After removal of duplicates, all retrieved articles were screened independently by two reviewers based on title and abstract. Studies that met the inclusion criteria were selected for full-text review, and discrepancies were resolved through discussion or consultation with a third reviewer. To ensure accuracy and consistency, standardized inclusion checklists were used during screening, and data extraction was cross-verified between reviewers before final entry. Extracted variables included author and year, study design, population characteristics, type and duration of PPI used, polypharmacy status, micronutrients assessed, assessment methods, and key outcomes. No automation tools were used in the selection or extraction process.

Risk of Bias Assessment

The risk of bias in included randomized controlled trials (RCTs) was assessed using the Cochrane Risk of Bias 2.0 (RoB 2) tool [9], which evaluates potential bias across five domains: randomization process, deviations from intended interventions, missing outcome data, measurement of the outcome, and selection of the reported result. Each domain was rated as having low risk, some concerns, or high risk, leading to an overall judgment of risk of bias. For the single prospective observational study included, the Newcastle-Ottawa Scale (NOS) [10] was applied, focusing on the selection of cohorts, comparability of groups, and outcome assessment. The results of these assessments were tabulated and interpreted in the context of overall study quality.

Data Synthesis and Analysis

Due to heterogeneity in study populations, PPI types, durations, outcome measures, and assessment methods, a meta-analysis was not performed. Instead, a qualitative synthesis of the findings was conducted. The narrative synthesis highlighted patterns of micronutrient deficiency, the strength of association with PPI use, and the consistency of findings across studies. Clinical implications and directions for future research were explored in detail in the discussion section.

Results

Study Selection Process

**Figure 1:**
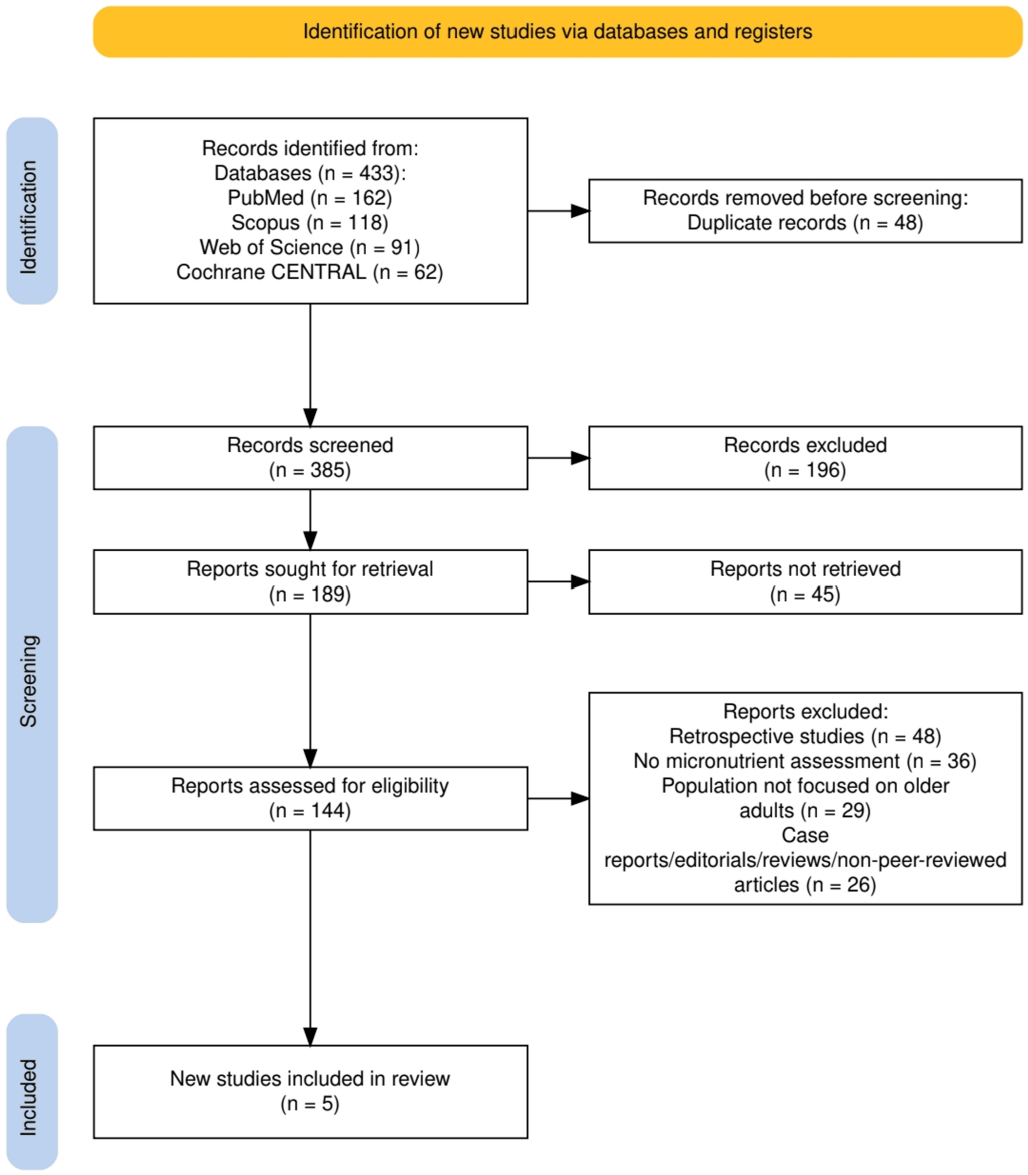
PRISMA flowchart illustrating the process of the selection of the studies. PRISMA: Preferred Reporting Items for Systematic reviews and Meta-Analyses

Figure 1 outlines the PRISMA flow diagram detailing the selection process of studies included in this review. A total of 433 records were identified through four databases: PubMed (162), Scopus (118), Web of Science (91), and Cochrane CENTRAL (62). After removing 48 duplicates, 385 records were screened, out of which 196 were excluded. Of the 189 full-text reports sought, 45 could not be retrieved. Among the 144 assessed for eligibility, 139 were excluded for reasons including retrospective design (48), lack of micronutrient assessment (36), non-older adult population (29), and being non-primary research (26). Ultimately, five studies met the inclusion criteria and were incorporated into the final review.

Characteristics of the Selected Studies

Table 1 summarizes the core characteristics of the five selected studies included in this systematic review. All studies were prospective in design, with the majority employing randomized controlled methodologies and varied durations of PPI exposure ranging from seven days to 12 months. The study populations included both healthy adults and individuals on concurrent medications such as diuretics and metformin, reflecting real-world polypharmacy scenarios. Micronutrients assessed encompassed vitamin B12, folate, magnesium, calcium, and related biomarkers like parathyroid hormone and bone turnover indicators. Assessment methods included biochemical assays, pharmacokinetic monitoring, and in one case, cognitive performance testing as an indirect indicator of nutrient status. Key findings varied, with some studies showing significant reductions in serum calcium and PTH levels, while others noted no change in magnesium or calcium absorption. Overall, the included studies demonstrate heterogeneity in population characteristics, intervention protocols, and outcome measures, reflecting the multifaceted nature of micronutrient dynamics in long-term PPI use.

**Table 1:** Summary of clinical trials evaluating the impact of long-term proton pump inhibitor use on micronutrient levels in older adults with polypharmacy. PPI: Proton Pump Inhibitor; RCT: Randomized Controlled Trial; MVMS: Multivitamin Multimineral Supplement; PTH: Parathyroid Hormone; BMD: Bone Mineral Density; PK/PD: Pharmacokinetics/Pharmacodynamics; TFCA: True Fractional Calcium Absorption; CANTAB: Cambridge Neuropsychological Test Automated Battery; P1NP: Procollagen Type 1 N-Terminal Propeptide; CTX: C-Terminal Telopeptide of Type I Collagen

Author, Year	Study Design	Population Characteristics	PPI Used (Type & Duration)	Polypharmacy Status	Micronutrients Assessed	Assessment Method	Key Outcomes
Chen et al., 2022 [11]	RCT (Double-blind, Placebo-controlled)	Men and women, 45–75 years; on diuretics, metformin, and/or PPIs	Not specified individually; part of inclusion (PPI use with other drugs); 16 weeks	Yes – All patients on at least one of: PPI, metformin, or diuretics	Vitamin B1, B12, C, folate, calcium, copper, magnesium, zinc	Fasting blood samples at baseline, 8 and 16 weeks	MVMS improved B12, folate, and vitamin C levels compared to placebo
Akter et al., 2015 [12]	RCT	60 healthy adult volunteers, age not specified; both genders; divided into six groups	Omeprazole, pantoprazole, lansoprazole, rabeprazole, esomeprazole; short-term (7 days)	Not specified	Vitamin B12 (linked indirectly to cognitive function)	Cognitive assessment via CANTAB software; no direct micronutrient assay	All PPIs impaired cognitive domains; omeprazole had most significant impact, esomeprazole the least
Bahtiri et al., 2017 [13]	Prospective Open-Label Comparative Study	250 adults with normal baseline magnesium and calcium; 209 completed 12-month follow-up	PPI duration: 12 months	Not specified	Magnesium, Calcium, PTH	Serum magnesium, total calcium, and PTH levels at baseline and 12 months	No change in magnesium; calcium and PTH levels decreased significantly over time
Hansen et al., 2019 [14]	RCT (Double-blind, Placebo-controlled)	115 healthy postmenopausal women, aged 45–75	Dexlansoprazole 60 mg, esomeprazole 40 mg daily; 26 weeks	Not specified	Calcium (via TFCA), PTH, Bone turnover markers (P1NP, CTX)	Serum and urine mineral levels, bone mineral density (BMD), PTH, and TFCA	No significant effect on calcium absorption, BMD, or PTH; bone turnover markers increased but remained within normal range
Kim et al., 2024 [15]	RCT (Open-label, crossover design)	49 healthy adult volunteers	Fixed-dose combo: esomeprazole 40 mg + magnesium hydroxide 350 mg vs. enteric-coated esomeprazole 40 mg; 7 days per treatment arm	Not specified	Magnesium (via co-formulation), Gastric pH (as absorption marker)	Pharmacokinetics, 24-hour gastric pH monitoring	Combo showed faster absorption and quicker gastric pH suppression; PK/PD equivalent to standard esomeprazole

Risk of Bias Assessment

The risk of bias assessment for the selected studies, as summarized in Table 2, reveals an overall moderate to low risk of bias across the included trials. Most RCTs demonstrated a low risk in key domains such as randomization, adherence to intended interventions, and outcome measurement, supporting the internal validity of their findings. However, one study showed some concerns due to limitations in blinding and reporting, which may influence interpretability. The prospective cohort study, assessed using the NOS, showed adequate outcome assessment but limited clarity in group comparability, leading to a moderate risk classification. These evaluations underscore the relative methodological strength of the included evidence while highlighting areas where reporting and design could be enhanced in future research.

**Table 2:** Risk of bias assessment of included studies using the RoB 2.0 and Newcastle-Ottawa Scale. RoB 2.0: Revised Cochrane Risk of Bias Tool for Randomized Trials; NOS: Newcastle-Ottawa Scale; RCT: Randomized Controlled Trial

Author, Year	Tool Used	Study Design	Randomization Process	Deviations from Intended Interventions	Missing Outcome Data	Measurement of the Outcome	Selection of the Reported Result	Overall Risk of Bias
Chen et al., 2022 [11]	RoB 2.0	RCT	Low risk	Low risk	Low risk	Low risk	Low risk	Low
Akter et al., 2015 [12]	RoB 2.0	RCT	Some concerns	Some concerns	Low risk	Some concerns	Some concerns	Some concerns
Bahtiri et al., 2017 [13]	NOS	Prospective Cohort	Representativeness: Adequate Comparability: Not clearly stated Outcome assessment: Adequate	–	–	–	–	Moderate (6/9 stars)
Hansen et al., 2019 [14]	RoB 2.0	RCT	Low risk	Low risk	Low risk	Low risk	Low risk	Low
Kim et al., 2024 [15]	RoB 2.0	RCT (crossover)	Low risk	Some concerns (open-label design)	Low risk	Low risk	Low risk	Some concerns

Discussion

Across the five included studies, a recurring pattern emerged linking long-term or repeated exposure to PPIs with alterations in micronutrient levels, particularly vitamin B12, folate, and calcium. Chen et al. demonstrated that in older adults receiving PPIs alongside other commonly prescribed medications such as diuretics and metformin, 16 weeks of multivitamin/multimineral supplementation led to significant improvements in serum folate (mean change +7.5 ng/mL vs. -1.6 ng/mL in placebo, p < 0.0001), vitamin B12 (+159.2 pg/mL vs. -33.9 pg/mL, p = 0.007), and vitamin C (+0.2 mg/dL vs. 0.0 mg/dL, p = 0.004), suggesting underlying deficiencies or increased requirements in this population [11]. Consistent with this, Akter et al. found that short-term use of PPIs resulted in measurable cognitive impairment in healthy adults, which the authors attributed, at least in part, to potential vitamin B12 depletion, most notably with omeprazole showing significant deficits in seven cognitive domains [12]. Calcium-related changes were also reported in two trials: Bahtiri et al. observed significant reductions in serum calcium and parathyroid hormone (PTH) levels over 12 months of PPI use in a large adult cohort [13], while Hansen et al., in a controlled trial of healthy postmenopausal women, noted no statistically significant change in calcium absorption or BMD despite elevated bone turnover markers such as P1NP and CTX [14]. Interestingly, while magnesium deficiency is a well-recognized risk of prolonged PPI use, Bahtiri et al. found no significant changes in serum magnesium after 12 months, a result potentially explained by normal baseline nutritional status and low-risk population [13]. In contrast, Kim et al. highlighted that a fixed-dose combination of esomeprazole and magnesium hydroxide produced faster gastric acid suppression than standard esomeprazole, but no direct conclusions on systemic magnesium status were drawn [15]. Collectively, these findings support a link between chronic PPI exposure and micronutrient depletion, particularly in at-risk populations, though inter-study variability suggests the need for more standardized and population-specific investigations. Importantly, these biochemical changes carry direct clinical implications, as unrecognized deficiencies may contribute to cognitive decline, bone fragility, and fall risk in older adults, underscoring the value of routine monitoring and targeted supplementation in long-term PPI users.

The clinical significance of the observed micronutrient alterations in long-term PPI users extends well beyond serum values; they reflect biologically and functionally relevant deficiencies that may exacerbate age-related health decline [16]. Vitamin B12 deficiency, for instance, is a well-established contributor to cognitive dysfunction, and its association with PPI use is mechanistically plausible given the dependence of B12 absorption on gastric acid-mediated cleavage from dietary proteins [17]. In older adults, who already experience physiological hypochlorhydria, PPI-induced suppression of gastric acid may further impair B12 bioavailability, leading to subtle cognitive impairments that accumulate over time. The study by Akter et al. underscores this, showing measurable deficits in cognitive domains even after short-term PPI use, likely mediated by subclinical B12 deficiency [12]. Similarly, calcium and magnesium absorption are both pH-dependent processes, and their diminished availability in the context of chronic acid suppression may increase the risk of osteopenia, osteoporosis, and subsequent fractures [18]. Reductions in serum calcium and PTH observed in Bahtiri et al.'s study [13] align with this concern, especially when considered alongside bone turnover marker elevations reported by Hansen et al. [14]. While the absolute changes may remain within reference ranges, such subclinical shifts could hold cumulative consequences in a geriatric population already prone to falls and bone fragility. Therefore, these micronutrient disruptions are not merely laboratory anomalies but may represent modifiable risk factors for serious geriatric syndromes.

Despite converging evidence suggesting a relationship between PPI use and micronutrient disturbances, heterogeneity across studies introduces complexity in drawing definitive conclusions. For instance, Bahtiri et al. reported no significant changes in serum magnesium after 12 months of PPI therapy [13], contradicting prior observational signals linking PPI use with hypomagnesemia. Several methodological limitations may account for this discrepancy: the study cohort consisted of relatively healthy individuals with normal baseline magnesium and calcium levels, dietary intake was not controlled or monitored, and the population's nutritional reserve may have masked subtle depletions. In contrast, studies like that of Chen et al. involved participants already receiving multiple medications known to impair nutrient absorption, such as metformin and diuretics, which could have compounded the effect of PPIs [11]. Additionally, sample sizes varied substantially across trials, with some studies including fewer than 60 participants and others lacking extended follow-up periods necessary to detect longer-term metabolic consequences. Moreover, most included trials excluded frail elderly individuals, institutionalized populations, or those with baseline malnutrition, all of whom are most susceptible to drug-nutrient interactions. These factors limit the generalizability of the findings and underscore the need for cautious interpretation of negative results.

Clinically, these findings highlight a pressing need to re-evaluate the long-term use of PPIs, especially in older adults exposed to polypharmacy [19]. While PPIs remain essential for managing acid-related disorders, chronic use without clear ongoing indications may predispose patients to avoidable micronutrient deficiencies with downstream health consequences. Routine nutritional screening, particularly for vitamin B12 and calcium, should be considered in older patients on long-term PPI therapy [4]. For individuals at higher risk, such as those with prior fractures, cognitive impairment, or multiple medication burdens, prophylactic supplementation may be warranted. Furthermore, clinicians should integrate deprescribing practices into routine care, assessing whether continued PPI use is clinically justified or whether step-down approaches (e.g., H2 receptor antagonists or on-demand therapy) may suffice [20]. Collaborative medication reviews involving pharmacists, dietitians, and geriatricians could be instrumental in reducing iatrogenic harm related to nutrient depletion. The cumulative impact of such micronutrient losses in aging adults may be subtle yet progressive, making early identification and intervention key to preserving function and reducing morbidity [21].

Looking ahead, future research should prioritize long-term, multicenter RCTs with adequately powered geriatric populations that reflect real-world complexity, including those with comorbidities, frailty, and poor nutritional status. Studies should go beyond biochemical endpoints and incorporate functional outcomes such as gait instability, cognitive decline, fracture rates, and hospital admissions. Furthermore, investigations should account for dietary intake, comedication use (especially metformin and diuretics), and socioeconomic factors that may influence both PPI prescribing patterns and nutritional risk [22]. There is also a need to explore the efficacy of mitigation strategies such as routine multivitamin supplementation or selective nutrient monitoring protocols. The role of deprescribing interventions in preventing micronutrient-related complications should be evaluated through implementation research in both primary care and institutional settings. Ultimately, bridging the gap between pharmacologic management and nutritional health will be critical for optimizing outcomes in the growing population of older adults exposed to long-term acid suppression therapy [23].

## Conclusions

This systematic review highlights the emerging concern of micronutrient deficiencies associated with long-term PPI use in older adults, particularly those burdened with polypharmacy. Across multiple clinical trials, consistent evidence suggests that chronic acid suppression may impair the absorption of key micronutrients, most notably vitamin B12, folate, and calcium, with potential consequences ranging from cognitive decline to compromised bone health. While some studies reported neutral outcomes, methodological differences and population characteristics likely influenced these results, underscoring the need for caution in interpretation. The significance of our study lies in its focused synthesis of clinical trial evidence, emphasizing not only the biological plausibility of PPI-induced nutrient deficits but also their relevance to real-world geriatric care. These findings call for heightened clinical awareness, routine nutritional monitoring, and judicious PPI prescribing practices, particularly in elderly individuals on multiple medications. By drawing attention to this often-overlooked aspect of chronic PPI therapy, our review advocates for a more integrated and preventive approach to medication management in aging populations.

## References

[1] Shanika LG, Reynolds A, Pattison S, Braund R (2023). Proton pump inhibitor use: systematic review of global trends and practices. Eur J Clin Pharmacol.

[2] Strand DS, Kim D, Peura DA (2017). 25 years of proton pump inhibitors: a comprehensive review. Gut Liver.

[3] Valdovinos-García LR, Villar-Chávez AS, Huerta-Iga FM (2025). Good clinical practice recommendations for proton pump inhibitor prescription and deprescription. A review by experts from the AMG. Rev Gastroenterol Mex (Engl Ed).

[4] Heidelbaugh JJ (2013). Proton pump inhibitors and risk of vitamin and mineral deficiency: evidence and clinical implications. Ther Adv Drug Saf.

[5] Heuberger RA, Caudell K (2011). Polypharmacy and nutritional status in older adults: a cross-sectional study. Drugs Aging.

[6] Ghazzawi HA, Hussain MA, Raziq KM (2023). Exploring the relationship between micronutrients and athletic performance: a comprehensive scientific systematic review of the literature in sports medicine. Sports (Basel).

[7] Page MJ, McKenzie JE, Bossuyt PM (2021). The PRISMA 2020 statement: an updated guideline for reporting systematic reviews. BMJ.

[8] Brown D (2020). A review of the PubMed PICO tool: using evidence-based practice in health education. Health Promot Pract.

[9] Sterne JA, Savović J, Page MJ (2019). RoB 2: a revised tool for assessing risk of bias in randomised trials. BMJ.

[10] Stang A (2010). Critical evaluation of the Newcastle-Ottawa scale for the assessment of the quality of nonrandomized studies in meta-analyses. Eur J Epidemiol.

[11] Chen O, Rogers GT, McKay DL, Maki KC, Blumberg JB (2022). The effect of multi-vitamin/multi-mineral supplementation on nutritional status in older adults receiving drug therapies: a double-blind, placebo-controlled trial. J Diet Suppl.

[12] Akter S, Hassan MR, Shahriar M, Akter N, Abbas MG, Bhuiyan MA (2015). Cognitive impact after short-term exposure to different proton pump inhibitors: assessment using CANTAB software. Alzheimers Res Ther.

[13] Bahtiri E, Islami H, Hoxha R (2017). Proton pump inhibitor use for 12 months is not associated with changes in serum magnesium levels: a prospective open label comparative study. Turk J Gastroenterol.

[14] Hansen KE, Nieves JW, Nudurupati S, Metz DC, Perez MC (2019). Dexlansoprazole and esomeprazole do not affect bone homeostasis in healthy postmenopausal women. Gastroenterology.

[15] Kim Y, Bae S, Jeon I (2024). Pharmacokinetics and pharmacodynamics of a fixed-dose combination of esomeprazole and magnesium hydroxide compared to the enteric-coated esomeprazole. Clin Ther.

[16] Vinke P, Wesselink E, van Orten-Luiten W, van Norren K (2020). The use of proton pump inhibitors may increase symptoms of muscle function loss in patients with chronic illnesses. Int J Mol Sci.

[17] Mumtaz H, Ghafoor B, Saghir H (2022). Association of vitamin B12 deficiency with long-term PPIs use: a cohort study. Ann Med Surg (Lond).

[18] Ito T, Jensen RT (2010). Association of long-term proton pump inhibitor therapy with bone fractures and effects on absorption of calcium, vitamin B12, iron, and magnesium. Curr Gastroenterol Rep.

[19] Maes ML, Fixen DR, Linnebur SA (2017). Adverse effects of proton-pump inhibitor use in older adults: a review of the evidence. Ther Adv Drug Saf.

[20] Farrell B, Pottie K, Thompson W (2017). Deprescribing proton pump inhibitors: evidence-based clinical practice guideline. Can Fam Physician.

[21] Yaacob NL, Loganathan M, Hisham NA, Kamaruzzaman H, Isa KA, Ibrahim MI, Ng KW (2024). The impact of pharmacist medication reviews on geriatric patients: a scoping review. Korean J Fam Med.

[22] Alshakhs S, Mohamed S, Kamal I, Laws S, Mahmoud MA (2025). The scope of frailty assessment tools in the Middle East: unraveling gaps and trends. Arch Gerontol Geriatr Plus.

[23] Haridas S, Ramaswamy J, Natarajan T, Nedungadi P (2022). Micronutrient interventions among vulnerable population over a decade: a systematic review on Indian perspective. Health Promot Perspect.

